# *Prickle1* mutation causes planar cell polarity and directional cell migration defects associated with cardiac outflow tract anomalies and other structural birth defects

**DOI:** 10.1242/bio.015750

**Published:** 2016-02-16

**Authors:** Brian C. Gibbs, Rama Rao Damerla, Eszter K. Vladar, Bishwanath Chatterjee, Yong Wan, Xiaoqin Liu, Cheng Cui, George C. Gabriel, Maliha Zahid, Hisato Yagi, Heather L. Szabo-Rogers, Kaye L. Suyama, Jeffrey D. Axelrod, Cecilia W. Lo

**Affiliations:** 1 Department of Developmental Biology, University of Pittsburgh School of Medicine, Pittsburgh, PA 15201, USA; 2 Department of Pathology, Stanford University School of Medicine, Stanford, CA 94305, USA; 3 Department of Oral Biology, University of Pittsburgh School of Dental Medicine, Pittsburgh, PA 15261, USA

**Keywords:** Biliary atresia, Cell polarity, Outflow tract, *Prickle1*

## Abstract

Planar cell polarity (PCP) is controlled by a conserved pathway that regulates directional cell behavior. Here, we show that mutant mice harboring a newly described mutation termed *Beetlejuice* (*Bj*) in *Prickle1* (*Pk1*), a PCP component, exhibit developmental phenotypes involving cell polarity defects, including skeletal, cochlear and congenital cardiac anomalies. *Bj* mutants die neonatally with cardiac outflow tract (OFT) malalignment. This is associated with OFT shortening due to loss of polarized cell orientation and failure of second heart field cell intercalation mediating OFT lengthening. OFT myocardialization was disrupted with cardiomyocytes failing to align with the direction of cell invasion into the outflow cushions. The expression of genes mediating Wnt signaling was altered. Also noted were shortened but widened bile ducts and disruption in canonical Wnt signaling. Using an *in vitro* wound closure assay, we showed *Bj* mutant fibroblasts cannot establish polarized cell morphology or engage in directional cell migration, and their actin cytoskeleton failed to align with the direction of wound closure. Unexpectedly, *Pk1* mutants exhibited primary and motile cilia defects. Given *Bj* mutant phenotypes are reminiscent of ciliopathies, these findings suggest *Pk1* may also regulate ciliogenesis. Together these findings show *Pk1* plays an essential role in regulating cell polarity and directional cell migration during development.

## Abstract (precis)

**Summary:** Outflow tract malalignment and multiple birth defects observed in the *Prickle1* mutant may arise from cell polarity perturbation, which may involve disruptions in Wnt signaling and of cilia function.

## INTRODUCTION

Planar cell polarity (PCP) is an evolutionarily conserved pathway that plays an important role in development. PCP has been examined in the context of developmental patterning in the *Drosophila* wing imaginal disk and compound eye, and refers to the polarization of cells within an epithelial sheet, orthogonal to the apical-basal polarity axis. Genetic analysis in *Drosophila* identified a group of interacting core PCP components that includes Van Gogh/Strabismus, Prickle, Frizzled, Dishevelled, Diego, and Flamingo (Devenport, 2014; Lawrence et al., 2007; Vladar et al., 2009). These proteins accumulate in asymmetrically localized complexes at proximal and distal apical cell junctions where they establish molecular cell polarity along the forming tissue axes via cell-cell communication (Axelrod, 2009). These proteins are conserved in vertebrates, and mutations in them cause a wide spectrum of developmental anomalies (Wansleeben and Meijlink, 2011), including the misalignment of hair cells in the cochlea, neural tube closure, brain and skeletal defects, and congenital heart disease (Cui et al., 2013).

In addition to epithelial planar polarization, PCP has been shown to regulate convergent-extension movements required for tissue morphogenesis. In *Xenopus* and mouse embryos, PCP-driven convergent-extension controls tissue elongation during gastrulation and neural tube closure (Cui et al., 2011; Dady et al., 2014; Juriloff and Harris, 2012; Wallingford and Harland, 2002). PCP also has been suggested to regulate directional migration of neural crest cells. This may contribute to the cardiac and craniofacial developmental anomalies in animal models with mutations in PCP core components (Montcouquiol et al., 2003; Simons et al., 2005; Simons and Mlodzik, 2008; Tada and Smith, 2000). However, the role of PCP in neural crest cell migration has been questioned (Pryor et al., 2014). Mice with mutations in the core PCP genes *Vangl2*, *Scrib* (Phillips et al., 2007), and *Dvl 1*, *2*, and *3* (Etheridge et al., 2008; Hamblet et al., 2002; Sinha et al., 2012) exhibit a similar spectrum of cardiac phenotypes involving outflow tract malalignment and septation defects (Boczonadi et al., 2014; Henderson et al., 2006). These cardiac defects likely involve not only perturbation of cardiac neural crest (CNC) cells, which are required for outflow septation, but also the second heart field (SHF). SHF cells migrate into the developing heart tube, forming most of the outflow tract, a structure that is often impacted by PCP mutations (Cohen et al., 2007; Schlessinger et al., 2009; Verzi et al., 2005).

*Prickle1* (*Pk1*) is a PCP core component that has been shown to cause progressive monoclonic epilepsy-ataxis syndrome in human clinical studies (Tao et al., 2011). Studies in mice suggest *Pk1* also plays an important role in development. This is indicated by the finding of pregastrulation lethality of *Pk1* knockout mouse embryos (Tao et al., 2009). The recovery of a hypomorphic *Pk1^C251X^* mouse allele allowed survival to mid-gestation, making it possible to observe neural tube defects, cleft palate, and kidney defects, while heterozygote animals were used to model epilepsy (Liu et al., 2013; Sowers et al., 2014; Yang et al., 2014; Yates et al., 2010). Similarly, using a *Pk1* conditional allele, a spectrum of defects is observed that is described to phenocopy human Robinow syndrome with multiple organ system defects (Liu et al., 2014a).

In this study, we report findings from a novel *Pk1* missense allele, named *Beetlejuice* (*Bj*)*.* Unlike other *Pk1* mutants, the *Bj* mutant survives to term, exhibiting a wide spectrum of developmental anomalies that include congenital heart defect, skeletal and craniofacial anomalies, and cochlea defects. We also show for the first time, *Pk1* mutation can cause biliary ductal hypoplasia in the spectrum of defects seen with biliary atresia. We provide evidence of a common mechanism involving disturbance of cell polarity and polarized cell migration contributing to the broad spectrum of developmental anomalies in the *Bj* mutant. This is associated with the disruption of canonical and noncanonical Wnt signaling.

## RESULTS

**Fig. 1.**
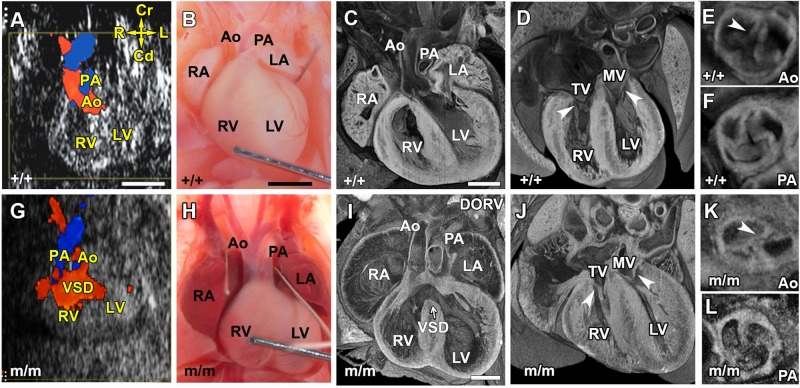
***Bj* mutant exhibits outflow tract malalignment defects.** (A,G) Echocardiography using color flow Doppler imaging showed in a normal E14.5 fetus (A), anterior positioning of blood flow from the aorta (Ao) emerging from the left ventricle (LV) and posterior positioning of blood flow from the pulmonary artery (PA) arising from the right ventricle (RV). In contrast, in the *Bj* mutant (G) fetus, blood flow streams from the outflow tract show parallel positioning, and this is associated with the mixing blood between the right ventricle and left ventricle, indicating aorta overriding the septum with a ventricular septal defect (VSD). Compass denotes orientation indicated by arrows; L, left; R, right; Cr, cranial; Cd, caudal. (B,H) Necropsy examination showed the aorta and pulmonary artery are abnormally positioned side by side in the mutant heart (H) compared to wild-type (B). RA, right atrium, LA, left atrium. (C,I) Further histopathology examination showed the *Bj* mutant aorta and pulmonary arteries are both connected to the right ventricle (RV), indicating it is a double outlet right ventricle (DORV) with a VSD (I). (D-F,J-L) The atrioventricular and semilunar valves are unaffected (J-L as compared to D-F, respectively; see arrowheads). TV, tricuspid valve; MV, mitral valve. Scale bars: 1 mm in A-H); 0.5 mm in C-L.

We recovered a novel mutant line, *Beetlejuice* (*Bj*), from a large ethylnitrosourea (ENU) mouse mutagenesis screen in C57BL/6J mice designed to interrogate the genetic etiology of human congenital heart disease (Liu et al., 2014b). Cardiovascular phenotyping was conducted using noninvasive fetal echocardiography to identify fetuses with structural heart defects. Color flow imaging by ultrasound biomicroscopy (UBM) showed in a wild-type fetus, two blood-flow streams that criss-cross (Fig. 1A), delineating the normal position of the two outflow tracts (Fig. 1B-D). In contrast, in a *Bj* mutant fetus, we observed regurgitant flow (Fig. 1G), indicating the presence of outflow tract malalignment in conjunction with abnormal blood flow showing the presence of a ventricular septum defect (VSD) between the two ventricular chambers (Fig. 1G). Together, this would suggest a congenital heart defect known as an overriding aorta (OA) or double outlet right ventricle (DORV) (Fig. 1H-J). In DORV, the aorta position is shifted rightwards to lie more than 50% over the right ventricle (RV), becoming aligned parallel to the pulmonary artery. When the shift is less than 50% over the RV, it is referred to as OA. Follow up necropsy examination confirmed parallel positioning of the aorta and pulmonary artery in the *Bj* mutants (Fig. 1H). Further histopathology by episcopic confocal microscopy (ECM) confirmed in *Bj* mutants, the diagnosis of a DORV with a perimembranous VSD (Fig. 1I vs wild type in Fig. 1C). Further examination of the cardiac valves showed the atrioventricular and semilunar valves (see arrowheads) in the *Bj* mutant heart (Fig. 1J-L) were indistinguishable from that seen in wild-type littermate control (Fig. 1D-F), indicating the OFT malalignment defect in the *Bj* mutant model is not related to defects in valvular morphogenesis. Systematic analysis of *Bj* mutants (*n*=29) showed 63% have CHD comprising a DORV, while 17% have an overriding aorta. In 20.6% (*n*=6), only a simple perimembranous VSD was observed with normally positioned great arteries (Table S1). In the course of this analysis, no inflow tract anomalies nor any type of laterality defects were ever observed.

To recover the disease causing mutation in *Bj* mutants, we mated the *Bj* mouse line to wild-type mice of a different inbred strain background (C57BL10) and conducted a genome scan in the hybrid mutant offspring using polymorphic (B6/B10) markers to map the CHD causing mutation. This analysis showed the mutation is situated in a 19 Mb interval on chromosome 15, between position 83,618,701 to the telomeric end of the chromosome (Table S2). To identify the pathogenic mutation, we carried out whole mouse exome sequencing analysis at 50× coverage using DNA from one mutant animal. Comparison of the sequencing data obtained from the *Bj* mutant vs the C57BL/6J reference genome identified three homozygous coding mutations (Tables S3, S4). Genotyping analysis of over 20 *Bj* mutants identified a missense mutation in *Pk1* (c.G482T:p.C161F) as the pathogenic mutation, as this mutation is the only one that is consistently homozygous in all of the Bj mutants. This missense mutation is in a highly conserved amino acid residue in the LIM1 domain, suggesting it is likely to be functionally important (Fig. S1). Consistent with this, analysis carried out using Polyphen-2, Provean, and Panther all predicted this mutation to be damaging or deleterious (Fig. S2) (Adzhubei et al., 2010; Brunham et al., 2005; Choi et al., 2012).

### Second heart field and cardiac neural crest perturbations

**Fig. 2.**
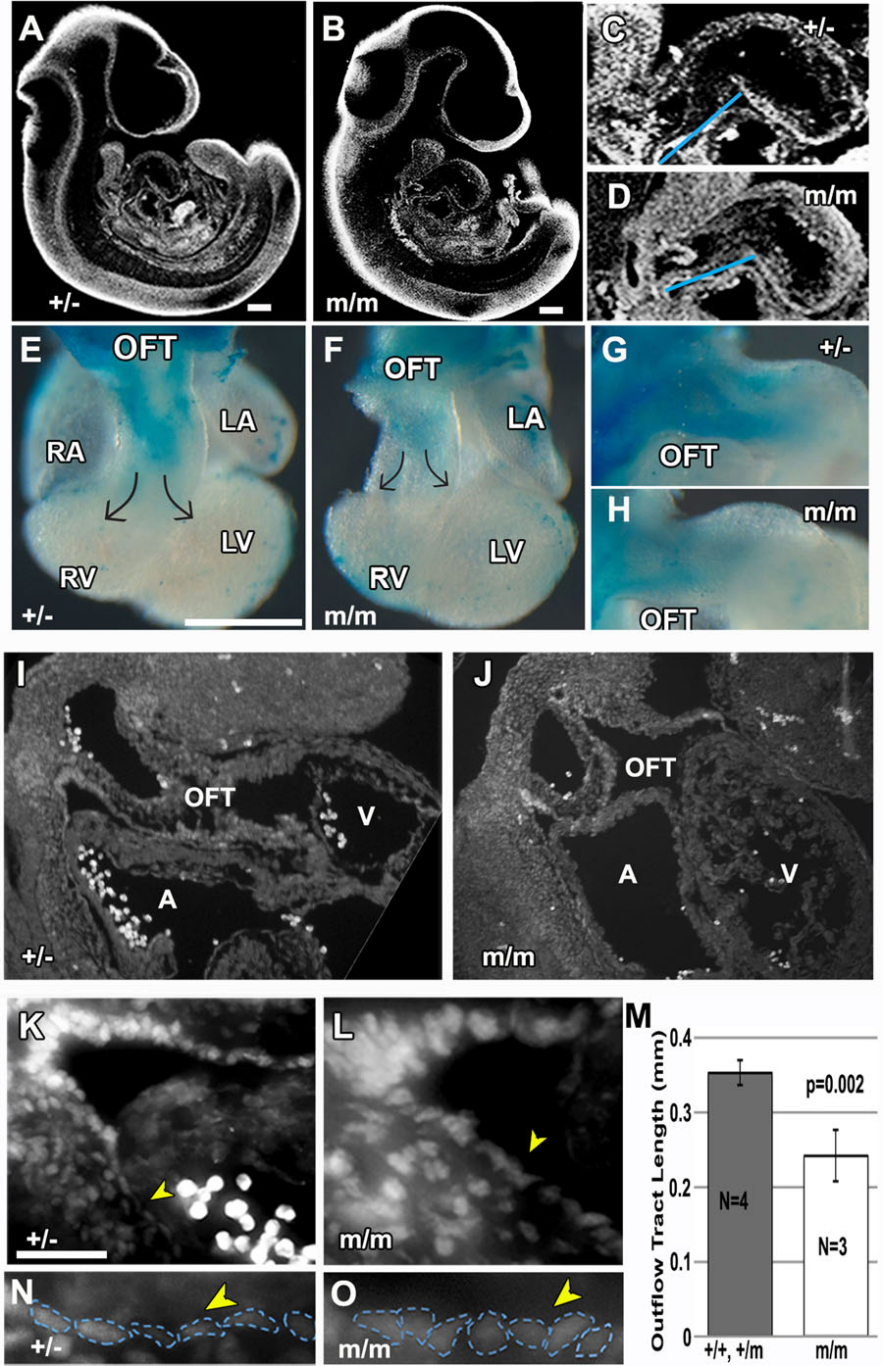
***Bj* mutants have shortened outflow tract and defects in neural crest and second heart field derivatives.** (A-D) Episcopic confocal histopathology in the sagittal plane of an E10.5 heterozygous (A) and *Bj* mutant (B) embryo is shown, with enlarged view of the outflow tract in (C) (*n*=4) and (D) (*n*=3) indicating reduction in length of the mutant OFT versus heterozygous (+/−) littermate control (see blue line in C,D). This was confirmed with further quantitative measurements (M). (E-H) Neural crest cells in the outflow tract of E10.5 embryos are visualized via a Cx43-*lacZ* transgene. This showed a reduction in neural crest cells in the homozygous *Bj* mutant heart (F,H) as compared to the heterozygous littermate control (E,G). (I-O) Sagittal sections of E10.5 embryo immunostained with anti-Islet1 (Isl1) antibody delineating second heart field cells in the dorsal pericardial wall (DPW) of the outflow tract of a wild-type (I,K) and *Bj* mutant embryo (J,L), with enlarged views shown in (K) and (L). Cells in the DPW wall (yellow arrowheads, denoted by blue outline in N,O) exhibit a flat squamous epithelial morphology in the wild-type embryo (N), but a distinct cuboidal morphology was observed in the homozygous *Bj* mutant embryo (O). A, atrium; V, ventricle. (M) Quantitative measurement using histopathology images showed a significant decrease in the length of the OFT in the homozygous *Bj* mutant hearts as compared to combined wild-type and heterozygous hearts. *P*-values were calculated with Student's *t*-test; error bars show standard deviation. Scale bars: 0.5 mm in A-D; 200 µm in E-H.

Development of the looped heart tube requires recruitment of second heart field (SHF) cardiac precursors that contribute to lengthening of the heart tube, eventually giving rise to much of the right ventricle, atria, and outflow tract (Cai et al., 2003; Kelly et al., 2001; Waldo et al., 2001). In embryonic day (E)10.5 *Bj* mutant embryos, we observed a significant reduction in OFT length (Fig. 2C vs D,M; *P*=0.002), suggesting possible defect in the recruitment of the SHF cells. Antibody staining with Islet1, a SHF marker (Cai et al., 2003), showed the splanchnic mesoderm in the dorsal posterior wall (DPW) and transition zone (TZ), regions contiguous to the distal end of the OFT, are comprised of SHF cardiac precursors (Fig. 2I-L). This is observed similarly in both the mutant and wild-type embryos. However, while these Islet positive SHF cells exhibited a flat squamous epithelial morphology in control embryos, a distinct change to a cuboidal cell shape was observed in the *Bj* mutants (Fig. 2N,O). This suggested a possible convergent-extension defect that may play contribute to shortening of the OFT.

We also examined whether there may be changes in the distribution of cardiac neural crest cells (NCC), as CNC deficiency can also cause OFT defects (Kirby et al., 1983). For this analysis, we intercrossed into the *Bj* mutant line, a Cx43 promoter driven *lacZ* reporter transgene previously shown to label neural crest cells (Lo et al., 1997). X-gal staining of E10.5 heterozygous control embryos (Fig. 2E,G) and *Bj* mutant embryos (Fig. 2F,H) showed a reduction in CNC cells in the OFT of the *Bj* mutant embryos, indicating the *Pk1* mutation may have disrupted CNC migration into the OFT. The use of heterozygous embryos as controls is appropriate as heterozygous *Bj* mice are viable and indistinguishable from wild-type animals.

### Disruption of epithelial cell polarity in second heart field cardiac precursors

**Fig. 3.**
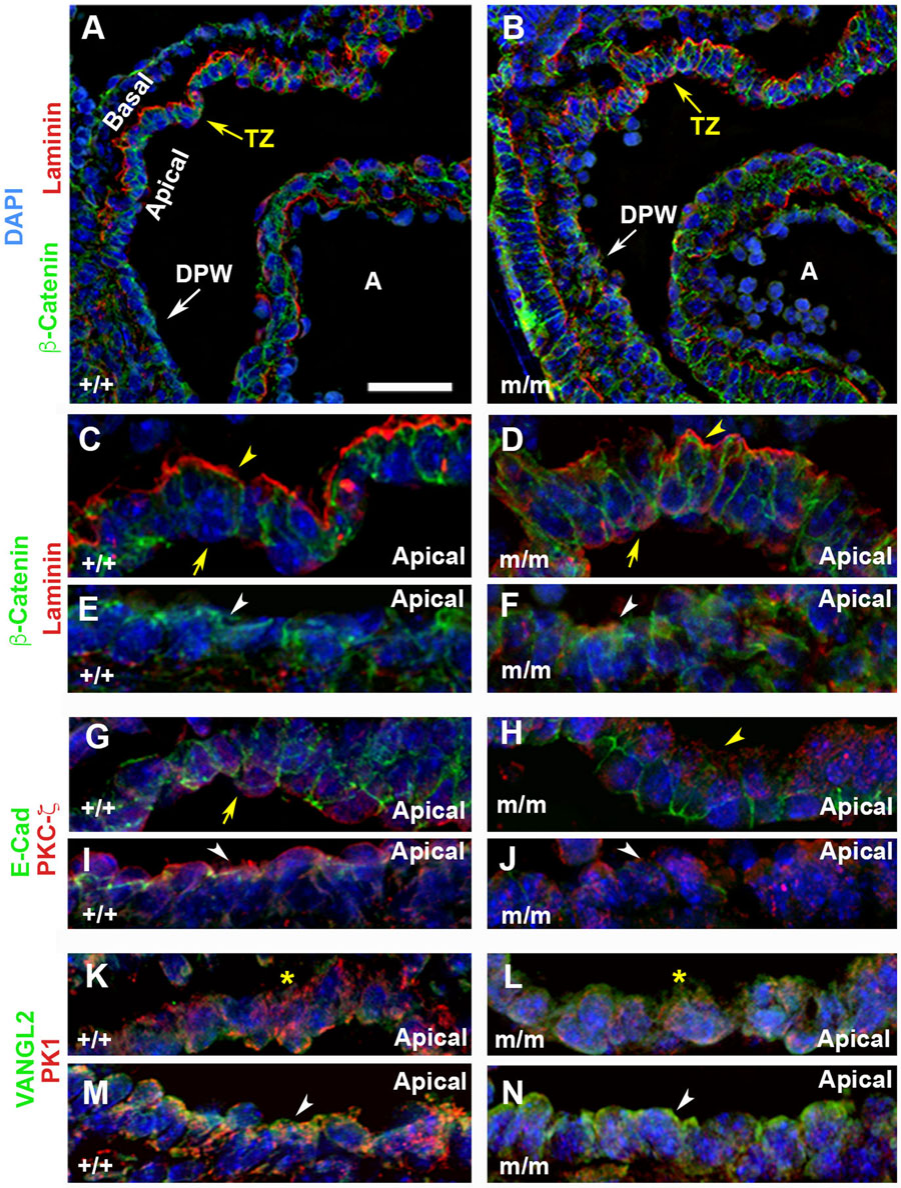
**Disruption of epithelial integrity in the developing outflow tract of the *Bj* mutant embryos.** Immunostaining with antibodies against β-catenin (green) and laminin (red) (A-F), and E-cadherin (green) and PKCζ (red) (G-J), and Vangl2 (green) and Pk1 (red) (K-N) of wild-type (A,C,E,G,I,K,M) (*n*=3), and *Bj* mutant embryos (B,D,F,H,J,L,N) (*n*=3) showed marked disorganization of the epithelium in the transition zone (TZ) and dorsal pericardial wall (DPW) of the E10.5 *Bj* mutant embryo. Panels C,E and D,F are enlarged views of A and B, respectively. Panels G-J and K-N are immunostaining of the same zones of A and B, respectively. Confocal imaging showed β-catenin is distributed along the cell surface in the control and *Bj* mutant embryos. Laminin is localized basally (arrowhead C,E) in the TZ of the control embryo, but in the mutant embryo, it is localized apically (arrow) and basally (arrow head D,F). While no laminin is detected in the DPW of the control embryo, it is ectopically expressed in the DPW of the *Bj* mutant embryo (compare E versus F). E-cadherin is widely expressed at regions of cell-cell contact, both in the TZ and in the DPW (G,I) of the control embryo. However, in *Bj* mutant embryos, E-cadherin expression is markedly reduced or extinguished in these regions (H,J). PKC-ζ expression is restricted to the apical (arrow) membrane of cells in the TZ and DPW epithelia (G,I). This polarized distribution is lost in the *Bj* mutant embryo, indicating the disruption of the epithelial organization of the DPW (H,J). Arrowheads in G-J indicate E-Cad and PKC-ζ distribution in the TZ and DPW. In the control embryo, Vangl2 and Pk1 are both expressed in the TZ and DPW of the outflow tract, with extensive co-localization observed (K,M). While Vangl2 expression is retained in the TZ and DPW of the *Bj* mutant, Pk1 expression is reduced (L,N; *n*=4; *P*=0.044). This is associated with an increase in overall thickness of the DPW. Arrowheads in M,N indicate Pk1 expression in the DPW, asterisks in K,L indicate Pk1 expression in TZ. Scale bar: 50µm in A-N. The *P* values were calculated with Student's *t*-test.

To identify the basis for the altered cell morphology in the SHF cardiac precursors in the DPW and TZ, we examined the expression of various epithelia cell markers in the OFT of E10.5 embryos using confocal microscopy. This analysis showed no change in the cell surface localization of β-catenin in the DPW and TZ of the *Bj* mutant embryos (Fig. 3B,F vs A,E). This would suggest canonical Wnt signaling was not affected. Examination of laminin expression showed in the TZ, the presence of ectopic laminin localized apically, while laminin expression was lost in some cells basally (see Fig. 3C as compared to wild type in Fig. 3D). Laminin is normally not expressed in the DPW (Fig. 3E), but ectopic expression was observed in the *Bj* mutant embryo (Fig. 3F). Further examination for expression of PKC-ζ, a protein kinase required for oriented cell division (Whyte et al., 2010), showed disruption of the normal apical localization observed in the TZ and DPW (Fig. 3G,I). In the *Bj* mutant embryo, little or no PKC-ζ expression was observed apically, but low level PKC-ζ expression was observed basally (Fig. 3H,J).

Together, these findings show the normal epithelial organization of the TZ and DPW of the OFT is disrupted, with the pseudostratified epithelial architecture of the DPW transformed into a thickened and disorganized multi-cell layered structure. To examine whether these changes may involve the perturbation of other PCP core components, we further examined the expression of Vangl2 and Scribble (Scrib). Both exhibited the same pattern of apical localization in the mutant and wild-type embryos (Fig. S3). We also examined the expression of Pk1 using an affinity purified anti-Pk1 antibody (see Fig. S4), and surprisingly, found Pk1 was markedly reduced in the *Bj* mutant embryo (Fig. 3L,N). This was confirmed with quantitative analysis of the Prickle1 immunofluorescence (*P*=0.044; mean intensity±standard deviation of 1.078±0.294 in wild-type vs 0.508±0.33 in homozygote mutant outflow tract; *n*=4). These findings suggest Pk1 is not required for specifying apical-basal cell polarity, but is required for the maintenance of epithelial tissue architecture in the TZ and DPW of the OFT.

### Perturbation of Wnt signaling in *Prickle1* mutants

**Fig. 4.**
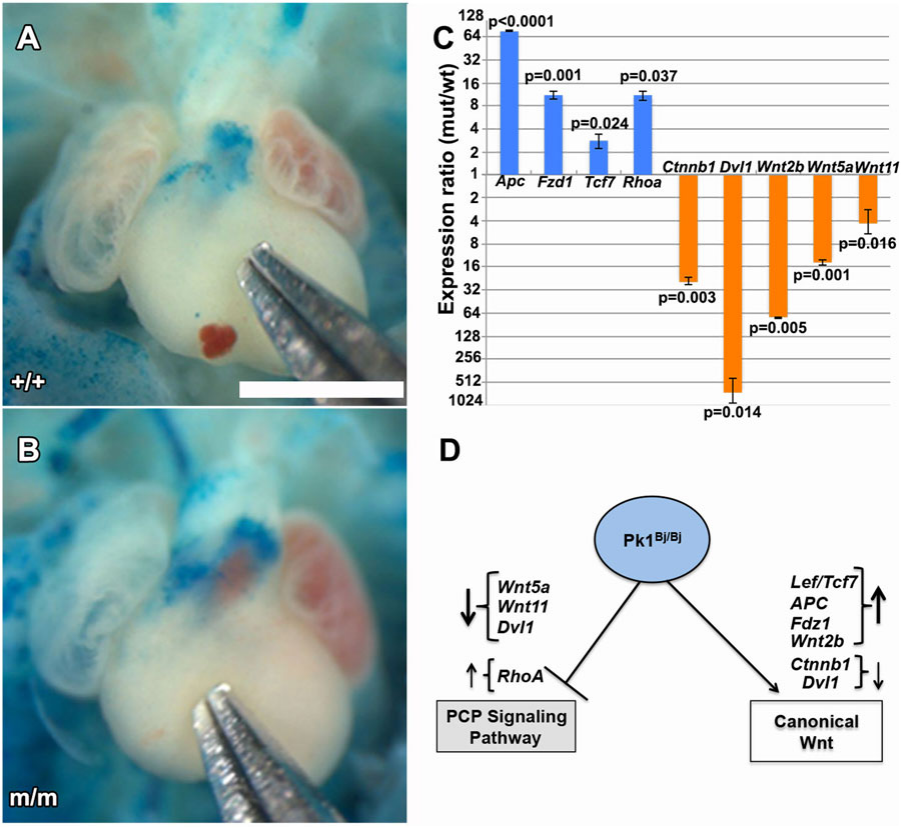
***Bj* mutants show disruption of canonical Wnt signaling.** (A,B) Analysis using the *BAT-lacZ* reporter showed increased *lacZ* reporter gene expression at the base of the outflow tract in the E13.5 *Bj* mutant heart (B) as compared to wild-type control (A). This suggests Wnt signaling is upregulated in the *Bj* mutant heart. Quantitative real-time PCR analysis (C) using RNA obtained from tissue isolated from the base of the OFT revealed changes in the expression of genes in both the canonical and noncanonical Wnt signaling pathway (D). Scale bar: 1 mm in A,B. The *P* values were calculated with Student's *t*-test; error bars show standard deviation.

Given Wnt signaling is known to play a role in regulating OFT development (Cohen et al., 2012; Schleiffarth et al., 2007), we examined whether the OFT defects in the *Bj* mutant embryo may involve perturbation in Wnt signaling. For this analysis, we made use of the canonical Wnt *BAT-lacZ* reporter*,* crossing it into the *Bj* mutant mouse line and using X-gal staining to assay for canonical Wnt signaling. This analysis revealed a marked increase in *lacZ* expression at the base of the outflow tract in the *BJ* mutant heart, indicating increased canonical Wnt signaling (Fig. 4A,B). Quantitative real-time PCR analysis (Fig. 4C) was carried out using cDNA made from RNA obtained from the base of the OFT where *BAT-lacZ* expression was observed to be elevated. This analysis showed gene expression changes that suggested the perturbation of both canonical (*Ctnnb1*, *Apc*, *Tcf7*, *Wnt2b*) and noncanonical (*Wnt5a*, *Wnt11*, *RhoA*) Wnt signaling in the *Bj* mutant heart (Fig. 4C,D). These changes are surprising and perhaps reflect some type of feedback regulation.

### Abnormal myocardialization in the *Bj* mutant

**Fig. 5.**
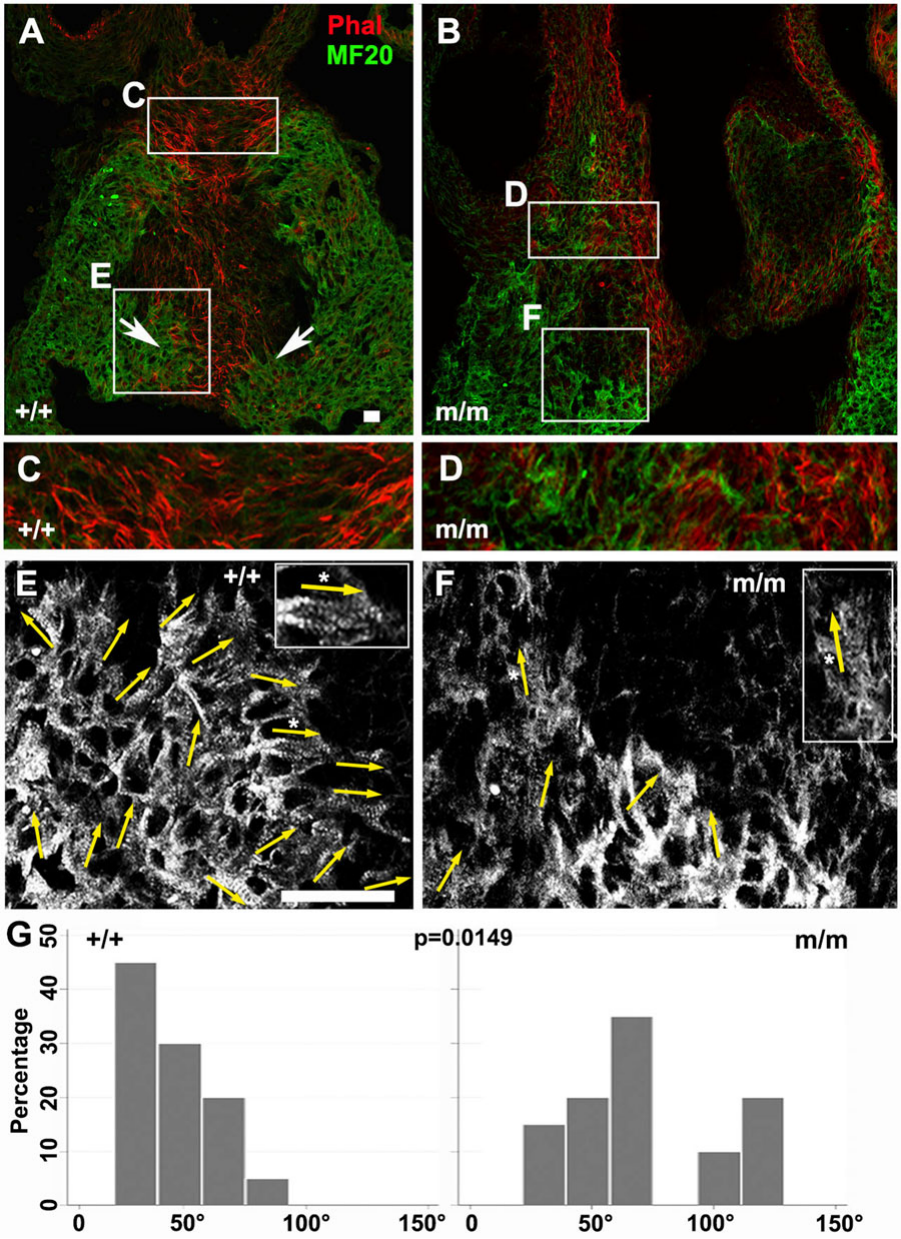
**Cell polarity and myocardialization defects in the OFT of *Bj* mutants.** (A-F) Phalloidin (red) and MF20 (green) staining of paraffin sections of wild-type (A,C,E,) (*n*=3) and *Bj* mutant (B,D,F,) (*n*=3) heart. In the wild-type heart, phalloidin staining showed actin filament alignment with the direction of cell invasion into the OFT cushion (A,C), but in the *Bj* mutant, this pattern of actin alignment is not observed (B,D). Arrows in A indicate the two prongs of cardiomyocytes invading the cardiac septum. Examination of the striated banding pattern from the MF20 immunostain showed the developing myofilaments are closely aligned and oriented towards the direction of myocardialization in the wild-type embryo (arrows, E), but in the *Bj* mutant, the myofilaments are sparse and are largely oriented perpendicular to the direction of myocardialization and septum formation (arrows, F). (G) Quantitation of myofilaments showed wild-type (*n*=20) cardiomyocytes are polarized in the direction of cell invasion; in *Bj* mutant hearts (*n*=20), the cell orientation is not properly aligned (*P*=0.0149). Quantitative analysis showed cardiomyocytes (MF20 positive) in this conotruncal region of the heart is significantly reduced compared to controls (+/+, +/m 70%; m/m 22%: *P*=0.0025). Scale bar: 20 µm. The *P* values were calculated with a two-sample Wilcoxon rank-sum test.

PCP has been shown to play a role in modulating cardiomyocyte migration to muscularize the outflow septum (Cui et al., 2013), a process referred to as myocardialization, that occurs after OFT septation (van den Hoff et al., 2001, 1999). Using MF20 immunostaining in the E13.5 wild-type mouse heart, the two prongs of invading myocardial cells can be visualized and are seen projecting into the outflow septum (see arrowheads in Fig. 5A). The direction of cardiomyocyte invasion is aligned with the orientation of actin filaments in the septal mesenchyme (visualized by phalloidin; Fig. 5A,C). Myofilaments in the invading cardiomyocytes are largely aligned with the direction of cell migration in the wild-type heart (asterisk Fig. 5E, inset in 5E). In contrast, in the E13.5 *Bj* mutant heart, the myocardial prongs are largely absent, and myofilaments in the cardiomyocytes are not well aligned with the direction of myocardialization (asterisk Fig. 5F, inset in 5F). Quantitative analysis showed a significant loss of oriented myofilaments alignment (Fig. 5G; *P*=0.0149). Phalloidin staining also revealed random orientation of actin filaments in the septal mesenchyme (Fig. 5D vs C). Together, these observations suggest that *Bj* mutants may fail to undergo normal myocardialization of the outflow septum due to a PCP defect that disrupts polarized alignment and migration of cardiomyocytes.

### Craniofacial defects and cranial neural crest perturbation

*Bj* mutants exhibited various extracardiac defects that included skeletal anomalies and craniofacial defects (Fig. S5). The craniofacial defects included micrognathia, hypoplastic frontal bones, and shortened snouts. *Bj* mutants also had shortened limbs. This was confirmed by quantitative analysis of skeletal preparations, which showed reductions in the length of the long bones and metacarpals (Fig. S5). Histological analysis showed that chondrocytes in the growth plate failed to align along the proximal-distal axis, likely contributing to the reduction in skeletal outgrowth. This contrasts with the well-organized chondrocytes aligned in columns along the long axis of limb outgrowth in wild-type embryos (Fig. S6). We also examined the distribution of cranial neural crest cells in E10.5 *Bj* mutant versus wild-type embryos using the Cx43-*lacZ* transgene, as neural crest cells play essential roles in craniofacial development (Minoux and Rijli, 2010). This analysis showed a decrease in neural crest cells in the mandibular and maxillary prominences of the *Bj* mutant embryos, suggesting neural crest perturbation may contribute to the craniofacial defects observed in *Bj* mutants (Fig. S7).

### PCP defects in the Cochlea

**Fig. 6.**
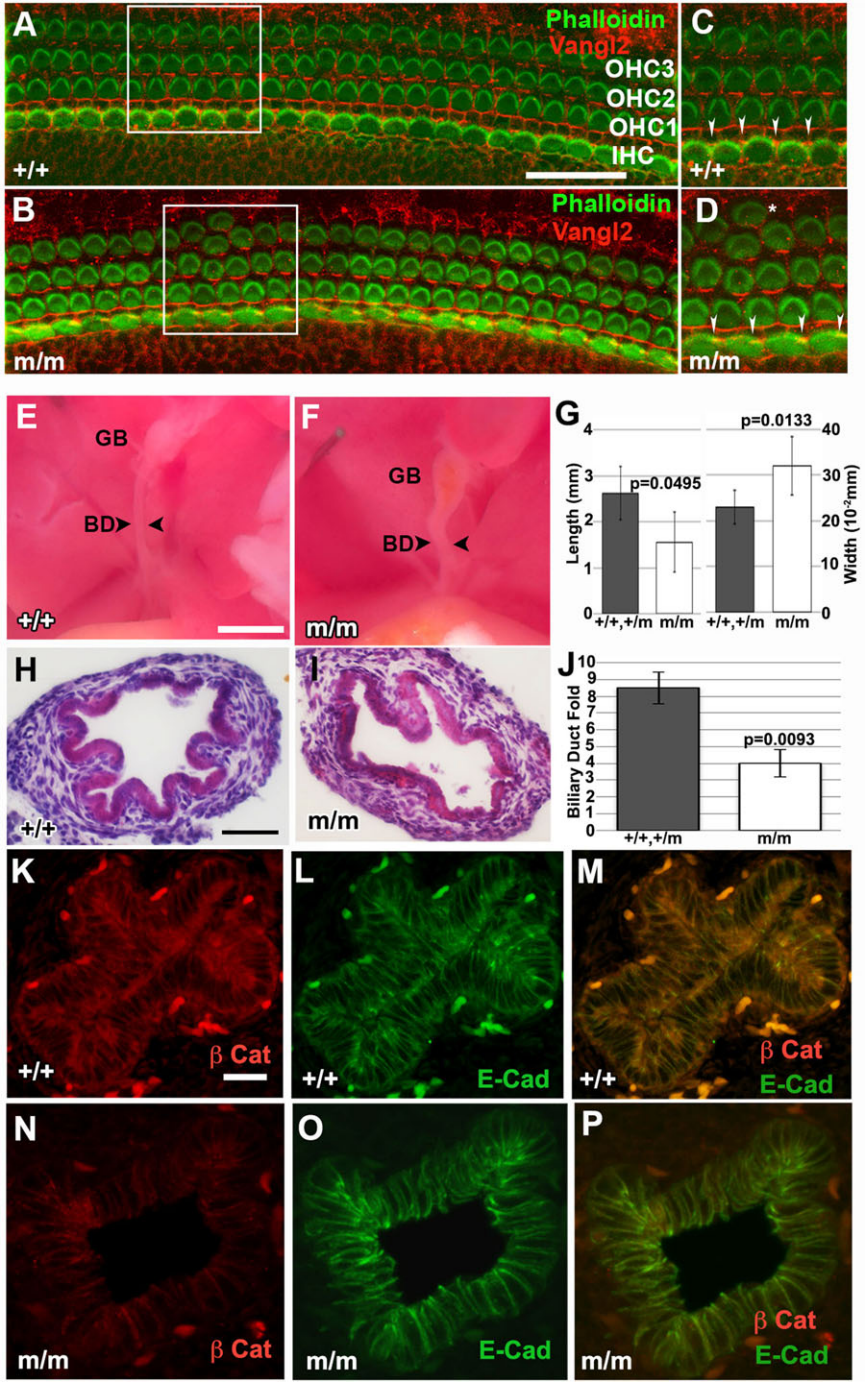
***Bj* mutants show stereocilia patterning defects and biliary duct (BD) malformations.** (A-D) Immunostaining with phalloidin (green) and Vangl2 (red) of the cochlea of near-term wild-type (A,C) and *Bj* mutant (B,D) embryo. The phalloidin staining delineated the normal chevron-shaped stereocilia in the outer hair cells (OHCs), showing malalignment in the *Bj* mutant (boxed B) compared to wild-type (boxed A). In addition, Vangl2 staining revealed a subtle misalignment of supporting cells connecting the inner hair cells (IHCs) in the *Bj* mutant (B), better seen in the enlarged views shown in panels C and D (see arrowheads). (E-J) Shown are bile ducts from a wild-type newborn mouse (E) (*n*=6) as compared to that of a *Bj* mutant (F) (*n*=6), which is noticeably shorter. Quantitative analysis showed a significant decrease in both the length and width of the *Bj* mutant biliary duct (G). Examination of hematoxylin and eosin stained sections of newborn wild-type (H) (*n*=4) and *Bj* mutant (I) biliary ducts suggest a decrease in the mucosal folds of the mutant duct (I) (*n*=5). This was confirmed with quantitative analysis, which showed a significant decrease in mucosal folds in the mutant duct (J). Error bars show standard deviation. (K-P) Immunostaining with antibodies against β-catenin (red) and E-cadherin (green) showed β-catenin expression is markedly reduced in the *Bj* mutant biliary duct (N,P), but no change was observed for E-Cadherin (O,P,). Scale bars: 100 µm in A-E); 30 µm (K-P). The *P* values were calculated with a two-sample Wilcoxon rank-sum test. GB, gallbladder; BD, bile duct.

We examined *Bj* mutants for cochlear defects using phalloidin staining to visualize the distribution of stereocilia and Vangl2 antibody labeling to assess molecular planar cell polarity. Normally, hair cells in the cochlea are arranged in repeating rows, with the actin-based stereociliary bundles exhibiting an identical polarized stereotypical chevron orientation, a patterning process that is PCP regulated (Fig. 6A,C) (Kelly and Chen, 2007). Analysis of three *Bj* mutant cochleae showed mild outer hair cell misalignment (Fig. 6B,D). While Vangl2 expression remained membrane localized in the supporting cells, the pattern of Vangl2 distribution was shifted, suggesting misalignment of the supporting cells adjacent to the inner hair cell (IHC) in the *Bj* mutant cochleae (Fig. 6D) compared to wild type (Fig. 6C). As this was observed in the basal, medial and apical regions of the cochlear duct, these changes may reflect global alternation in tissue architecture that could be elicited by a defect in convergent-extension cell movement.

### Biliary ductal abnormality

We examined the bile duct in the *Bj* mutant (Fig. 6F), and observed it was significantly shorter compared to wild type (Fig. 6E). Quantitative measurements revealed a decrease in the length, but an increase in width of the *Bj* mutant bile ducts (Fig. 6G). This was accompanied by reduction in the number of mucosal folds (Fig. 6H-J). Given several studies have suggested a role for β-catenin dependent canonical Wnt signaling in biliary fate determination (Tan et al., 2008), we conducted confocal immunohistology to examine the expression of both β-catenin and E-cadherin in the bile duct. In the wild-type bile duct, β-catenin and E-cadherin were highly expressed and show colocalization at the cell surface (Fig. 6K-M). In the *Bj* mutant, there was no change in the pattern of E-cadherin expression (Fig. 6O), but β-catenin expression was reduced (Fig. 6N,P). Further immunostaining was carried out to examine the distribution of laminin, Vangl2, PKC-ζ, and Pk1 in the mutant and wild-type biliary duct. This analysis showed the expression of laminin was unchanged while all three proteins were reduced compared to the wild-type bile duct, but the overall pattern of expression was unchanged (Fig. S8). These findings show *Pk1* is required for normal biliary duct morphogenesis, but this does not involve the modulation of apical-basal polarity.

### Planar cell polarity and polarized cell migration defect in *Bj* mutant MEFs

**Fig. 7.**
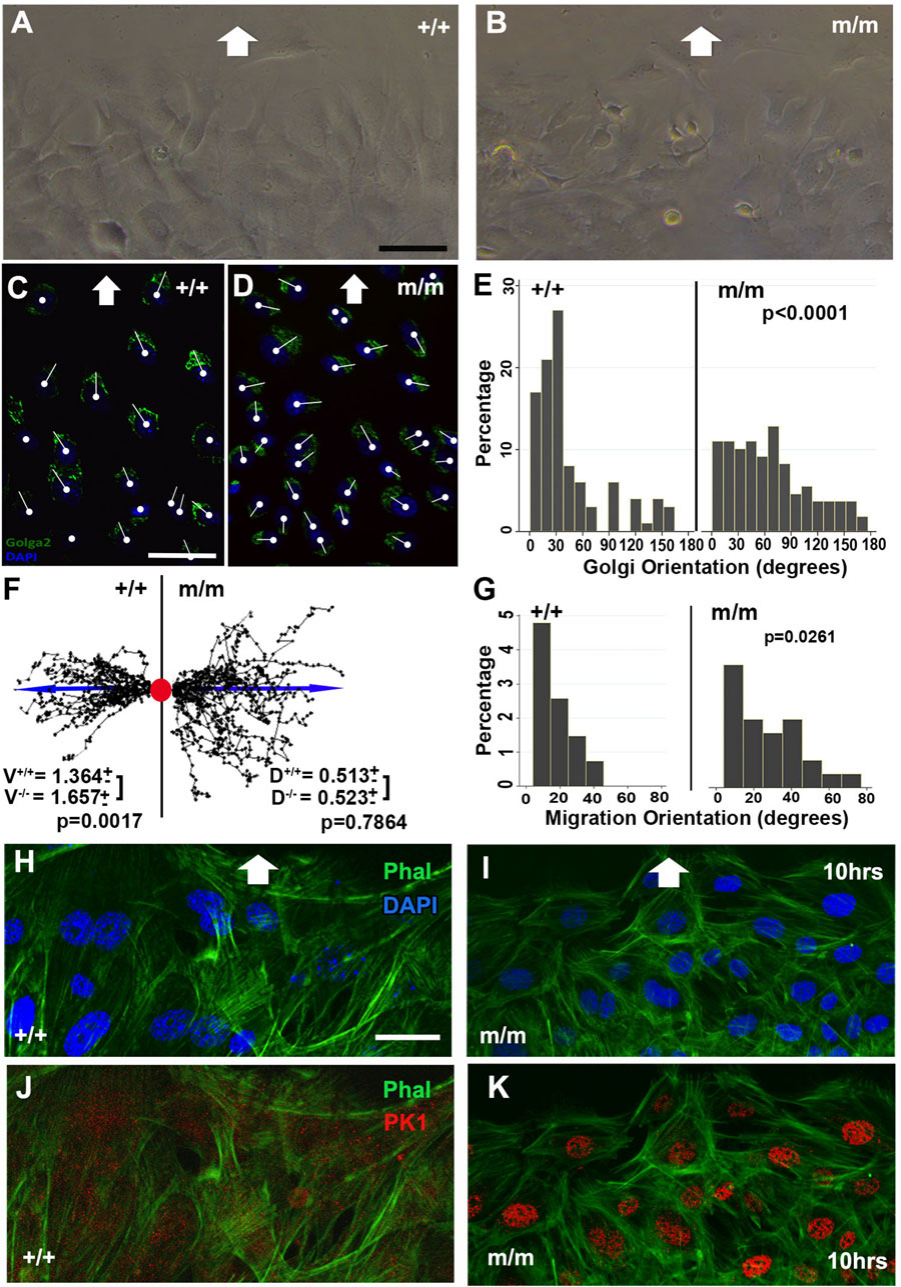
**Wound closure assay show defect in cell polarity and polarized cell migration in *Bj* mutant MEFs.** (A-G). Analysis of cell polarity and directional cell migration using a MEF wound closure assay. Wildtype MEFs (A) are aligned with the direction of wound closure (white arrows), but *Bj* mutant MEFs (B) exhibit randomized pattern of cell migration. Cell orientation is delineated by examining the Golgi position after 24 h observed by Golga2 (green) immunostaining (C,D). Golgi orientation is indicated by a white line drawn from the cell nucleus (white circle) through the center of the Golgi. (E-G) Quantitative analysis of Golgi orientation relative to the direction of cell migration showed Golgi orientation is polarized along the direction of cell migration in wild-type (*n*=100), but not *Bj* mutant MEFs (*n*=150) (E). Tracings of the migratory path of MEFs after 8 h showed straight migration paths for wild-type MEFs (*n*=28), but tortuous migration paths with increased velocity for mutant MEFs (*n*=27) (F,G) V, speed of cell locomotion; D, directionality of cell movement. (H-K) Phalloidin (green) and Pk1 (red) antibody staining of wild-type (H,J) and *Bj* mutant (I,K) MEFs migrating into a wound gap after 10 h. This showed well formed actin stress fibers aligned with the direction of cell migration in the wild-type MEFs (H,J), but in the *Bj* mutants MEFs, the actin cytoskeleton exhibited a cortical basket formation (I,K). Interestingly, wild-type MEFs showed Pk1 localization in the cytoplasm (J), but in *Bj* mutant MEFs, Pk1 is localized to the nucleus (K). The *P* values were calculated with a two-sample Wilcoxon rank-sum test. Scale bars: 50 µm in A-D; 30 µm in H-K.

Previous studies have shown that disruption of PCP components can perturb convergent-extension cell movements, and other types of directional cell migration. To examine the potential role of *Pk1* in modulating directional cell movement, we generated *Bj* mutant and wild-type MEFs for wound scratch assays. MEFS were grown to confluence, and a wound gap was created with a scratch in the monolayer. Wildtype MEFs quickly became aligned to the direction of wound closure, but the mutant MEFs remained randomly oriented (Fig. 7A,B). This was demonstrated quantitatively with analysis of Golgi orientation using a Golga2 antibody (Fig. 7C,D). In migrating wild-type MEFs, the Golgi is situated at the cell's leading edge, aligned with the direction of cell migration. This is indicated by Golgi positioning that is mostly less than 60° relative to the direction of cell migration in the wound gap (Fig. 7C,E). In contrast, in *Bj* mutant MEFs, the distribution of Golgi orientation was broadened, indicating randomization (Fig. 7D,E; *P*<0.0001). These results indicate that *Bj* mutant MEFs are unable to establish the cell polarity required for efficient directional cell migration.

To examine further how *Pk1* may regulate directional cell migration, we conducted time-lapse videomicroscopy over 8 h to track the migratory behavior of individual cells during wound closure. By tracing the migratory paths of individual cells in the time-lapse videos, we observed a relatively straight migratory path in the wild-type MEFs (Fig. 7F,G), while a more tortuous migratory path was observed for the *Bj* mutant MEFs (Fig. 7F,G). This pattern of cell migration in the *Bj* mutant MEFS was correlated with a significant increase in the speed of cell locomotion (V in Fig. 7F; *P*=0.0017), but no net change in the directionality of cell movement (D in Fig. 7F; *P*=0.7864).

To examine for possible cytoskeletal changes that may account for the defect in polarized cell migration after 10 h, we carried out phalloidin staining to visualize the actin cytoskeleton. Wild-type MEFs showed well-formed actin stress fibers aligned with the direction of cell migration (Fig. 7H,J). In contrast, in *Bj* mutant MEFs, the actin stress fibers were organized in a basket configuration around the entire cell cortex, indicating no specific polarized orientation (Fig. 7I,K). We also carried out immunostaining with a *Pk1* antibody to determine if changes in Pk1 distribution may contribute to the defects observed in directional cell migration in the *Bj* mutant MEFs. Pk1 localization was observed in the cytoplasm in wild-type MEFs (Fig. 7J), but in the *Bj* mutant MEFs, it was largely localized to the nucleus (Fig. 7K). Pk1 was previously shown to bind REST, a transcriptional repressor, and sequester it in the cytoplasm to relieve transcription repression (Shimojo, 2006; Shimojo and Hersh, 2003). These findings are reminiscent of a previous study showing abnormal nuclear localization of a human *Pk1* mutant protein known to cause myoclonus epilepsy (Bassuk et al., 2008).

### Primary and motile cilium defects in *Bj* mutant cells

**Fig. 8.**
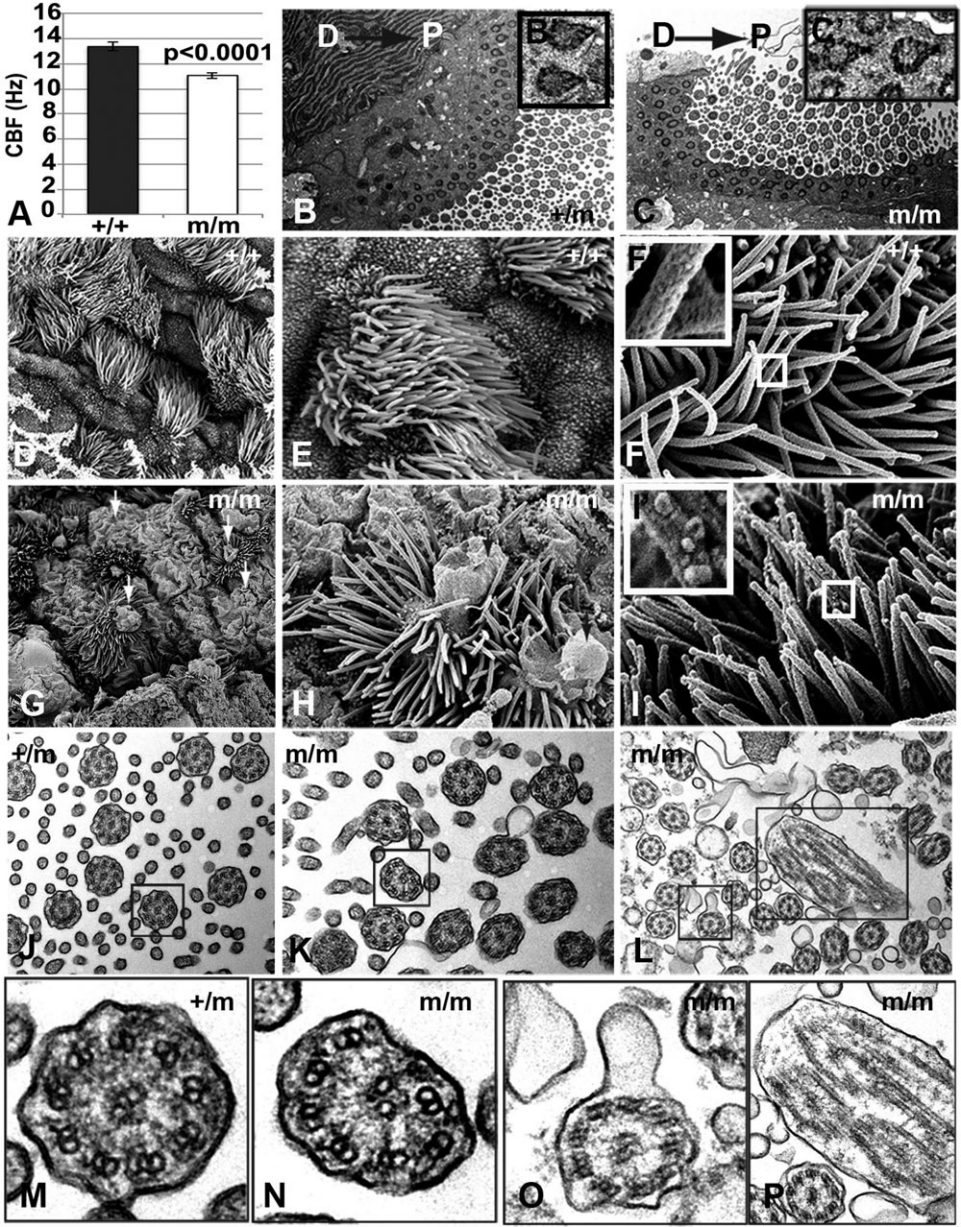
**Cilia defects in *Bj* mutant tracheae.** (A) Quantitative analysis shows a reduction in cilia beat frequency of *Bj* mutant tracheal airway cilia. Error bars show standard deviation. (B,C) Transmission electron microscopy (TEM) of motile cilia in both mutant (C) and control (B) trachea show normal planar polarity based on the orientation of basal feet (see boxed insets) on individual cilia towards the proximal (oral) direction. D, dorsal; P, proximal. (D-I). Scanning electron microscopy of *Bj* mutant trachea (bottom G,H) shows that compared to control (D,E) nonciliated and multiciliated cells have large apical membrane bulges (arrows G,H), which in the multiciliated cells incorporate the axonemes. (I) Some mutant ciliary axonemes also have blebbed surface (boxed I′). (J-L) TEM of *Bj* mutant cilia show some abnormal axonemal microtubules (K,L), membrane blebs and compound axonemes compared to control (J). (M-P) Enlarged views of (J-L) show the absence of a microtubule doublet in the mutant axoneme (N), blebbed membrane (O), and a compound axoneme (P) compared to the control (M). Compound axonemes are likely contained within the membrane blebs observed by SEM (G,H).

Given previous studies indicating a role for cilia in constraining canonical versus noncanonical Wnt signaling (He, 2008) we further examined ciliogenesis in *Bj* mutants MEFs. Cilia were visualized by immunostaining with antibodies to acetylated α-tubulin and γ-tubulin to mark the axoneme and basal body, respectively, after serum starvation to stimulate ciliogenesis. *Bj* mutant MEFs (Fig. S9A,B) exhibited a reduction in ciliogenesis (Fig. S9D; *P*=0.0304) and the cilia formed were shorter compared to wild-type MEFs (Fig. S9C; *P*<0.0001). To examine for possible defects in motile cilia, which have been shown to be aligned by PCP signaling (Vladar et al., 2012), we obtained the tracheal epithelia of newborn *Bj* mutants and littermate controls and conducted videomicroscopy to examine ciliary motion. Surprisingly, the *Bj* mutant exhibited a significant reduction in cilia beat frequency (Fig. 8A). Transmission electron microscopy (TEM) showed normal orientation of the basal feet of cilia in the *Bj* mutant tracheal epithelia as observed in littermate controls (Fig. 8B,C). Scanning electron microscopy (SEM) (Assemat et al., 2004) showed abnormal large apical membrane bulges that incorporated axonemes in the *Bj* mutant tracheal epithelium (Fig. 8G,H). SEM revealed membrane blebs at the surface of ciliary axonemes (Fig. 8I′, insets), while cilia in wild-type tracheal were smooth (Fig. 8F′, insets). Also observed were abnormal axonemal microtubules (Fig. 8K,L), and compound axonemes (Fig. 8J). Enlarged views (Fig. 8M-P) show absent microtubule doublets in some ciliary axonemes (Fig. 8N). Taken together, these results suggest *Pk1* may play a role in the regulation of primary and motile cilia structure and function.

## DISCUSSION

We showed the *Bj* mutant exhibits a wide spectrum of developmental anomalies that included abnormalities involving the cardiac OFT, biliary duct, cochlea and skeletal and craniofacial anomalies. We showed these defects arise from a missense mutation in *Pk1*, a PCP core component. Consistent with this, phenotypes observed in the *Bj* mutant are similar to those seen with mutations in other PCP core component (Etheridge et al., 2008; Hamblet et al., 2002). While no change was found in apical-basal polarity, we observed defects in planar cell polarity and directional cell migration. In the OFT, SHF cells formed a disorganized multilayered aggregate, indicating a defect in convergent extension cell movement required for delamination of a cohesive epithelial sheet mediating OFT lengthening (Sinha et al., 2012). A myocardialization defect was also observed with cardiomyocytes in the conotruncal region of the heart failing to align with the direction of cell invasion into the outflow cushion. This cell migration defect was associated with perturbation of both canonical and noncanonical Wnt signaling. These findings suggest the DORV phenotype in the *Bj* mutant may arise from the combined disruption of OFT lengthening and a myocardialization defect in the OFT.

Similar OFT malalignment defects also have been observed with mutations in several other PCP components (Henderson et al., 2006; Phillips et al., 2007; Sinha et al., 2012), indicating this pathway plays an important role in the pathogenesis of DORV. In the *Bj* mutant, OFT malformations were observed in conjunction with craniofacial and skeletal anomalies that included micrognathia and shortened limbs, phenotypes reminiscent of those seen in Robinow syndrome (Soman and Lingappa, 2015; Webber et al., 1990). Robinow is associated with mutations in *ROR2*, a WNT signaling component (Afzal et al., 2000a,b; Butler and Wadlington, 1987). *Wnt5a* and *Ror2* have been shown to regulate proteasomal degradation of Pk1, suggesting Pk1 acts downstream of Wnt5a (Liu et al., 2014a; Yang et al., 2014). However, in the *Bj* mutant, we observed expression of *Wnt5a* is reduced in the conotruncal heart tissue. This may reflect feedback regulation, or possible tissue specific differences in the integration of Pk1 and Wnt signaling. The craniofacial/skeletal and OFT defects in *Bj* mutants also are reminiscent of phenotypes associated with velocardiofacial (VCF) or DiGeorge syndrome (Digilio et al., 2005). Many of the phenotypes observed in VCF/DiGeorge syndrome are known to involve neural crest perturbation (Bockman and Kirby, 1984; Bockman et al., 1987; Scambler, 2000), consistent with our observation of abnormal deployment of cardiac and cranial neural crest cells in the *Bj* mutant.

We demonstrated *Bj* mutant MEFs were unable to establish cell polarity mediating directional cell migration. Analysis of cell migratory behavior showed decreased directionality, but increased speed of cell locomotion. The mutant MEFs, unlike wild-type MEFs, failed to form actin stress fibers aligned with the direction of cell migration. Furthermore, we observed myofilaments in cardiomyocytes of the OFT were not well aligned with the direction of cell migration, contributing to the myocardialization defect in *Bj* mutant heart. As the actin cytoskeleton is also known to play important roles in ciliogenesis (Kim et al., 2010; Yan and Zhu, 2013), it is significant to note that both motile and primary cilia defects were observed in the *Bj* mutant. Thus a decrease in ciliation and primary cilia length was observed in *Bj* mutant MEFs, while ciliary beat frequency was reduced in the tracheal epithelia. Basal foot positioning was unaffected, but ultrastructural defects were observed indicating detachment of the axoneme from the ciliary membrane. The latter finding is reminiscent of those reported in mice harboring a mutation in *Tbc1d32* (Ko et al., 2010), where cilia ultrastructural defects were also linked to disruption of the actin cytoskeleton. These findings suggest *Pk1* may have a role in regulating ciliogenesis, an intriguing possibility given the similarity in the developmental phenotypes observed in the *Bj* mutant and that of ciliopathy mutant mouse models. This is supported by our previous study showing *Wdpcp*, a known PCP effector, is required for ciliogenesis and modulation of the actin cytoskeleton required for the specification of cell polarity and directional cell migration (Cui et al., 2013).

Overall, these studies showed the *Pk1* mutation can cause a wide spectrum of structural birth defects with overlap to those seen in various ciliopathies and in Robinow and velocardiofacial syndromes. This is associated with defects in cell polarity, directional cell migration, and PCP regulated convergent extension cell movements (Rao Damerla et al., 2014). Further study is needed to investigate whether the cilia defects observed in the *Bj* mutant may reflect the known link between actin and the regulation of ciliogenesis, and if this may underlie the ciliopathy related birth defect phenotypes seen in PCP related mutants.

## MATERIALS AND METHODS

### Institutional approval for animal studies

All mouse experiments were conducted in accordance with animal protocols approved by the Institutional Animal Care and Use Committee at the University of Pittsburgh.

### Necropsy and histopathology examination for congenital heart disease

Newborn pups or E17.5 fetuses were collected, and fixed in 10% formalin. Necropsy examination was carried out to examine for external evidence of cardiac anomalies. To further evaluate for intracardiac defects, tissue was further analyzed by paraffin embedding and histopathology examination carried out using episcopic confocal microscopy (ECM). ECM imaging allows digital resectioning of the 2D serial image stack of the specimen and 3D reconstructions to uncover structural heart defects (Liu et al., 2014b).

### Recovery of the *Pk1* mutation and mouse breeding

Genomic DNA from line 019 (MGI: 5297388) mutant mice was sequence captured using Agilent SureSelect XT Mouse All Exon kit and sequenced with SOLiD 5500xl pair-end sequencing. Average 53.4× target coverage was achieved. Sequence reads were aligned to C57BL/6J mouse reference genome (mm9) and analyzed using LifeScope software (http://www.lifetechnologies.com). Sequence variants were annotated with ANNOVAR (www.openbioinformatics.org/annovar/) and custom scripts, and filtered against dbSNP and our in-house databases (Table S1). The three novel homozygous coding variants identified (Tables S2, S3) were then genotyped across all mutants from the same family. *Pk1^c.G482T:p.C161F^* was the only candidate mutation which was homozygous in all the mutants, thus indicating it is disease causing (Table S3).

### Mouse embryonic fibroblast isolation and analysis of primary ciliogenesis

Primary mouse embryonic fibroblasts (MEFs) were isolated from E12.5-E13.5 mutant embryos and their wild-type littermates as previously described (Cui et al., 2013). Briefly, embryos were minced using a sterile razor blade, 500 ml of 0.25% trypsin-EDTA was added, and samples were incubated at 37°C for 20 min. After incubation for 20 min in 10 cm tissue culture dishes, the remaining fragments were pipetted multiple times to dissociate cells and incubated overnight in DMEM supplemented with 10% FBS, with penicillin and streptomycin. For primary cilia analysis, MEFs were serum starved in DMEM supplemented with 0.1% or 0.25% serum for 36 to 48 h to induce cilium growth, respectively. Cells were fixed 4% paraformaldehyde in PBS and immunostained with acetylated antibodies against α-tubulin (Sigma T7451, 1:1000), γ-tubulin (Sigma T6557, 1:1000) (St. Louis, MO, USA), and Ift88 (gift from Dr Gregory Pazour, University of Massachusetts Medical School, USA). Cells were then imaged using an Olympus Fluoview FV1000 or a Nikon Eclipse Ti-inverted laser scanning confocal microscopes.

### Skeletal preparation and histo-morphometric analyses

Limbs of E15.5 and postnatal day (P)0 mice were fixed in 4% PFA at 4°C overnight and embedded in paraffin using standard protocols. For histomorphometry, 10-µm-thick longitudinal sections were cut through the forelimb and placed on SuperFrost Plus slides (Fisher Scientific 4951-001; Pittsburgh, PA, USA). For the wholemount skeleton staining to visualize bone and cartilage, E15.5 and P0 mice were fixed in 95% ethanol and stained with Alcian Blue/Alizarin Red. For visualization of the skull vault, neonates were euthanized and fixed in 4% paraformaldehyde overnight, cleared in 0.5% potassium hydroxide and stained in 0.1% Alizarin Red. Alcian Blue/Alizarin Red staining, and H&E, as well as Safranin O (shows maturation of cartilage in the growth plate) staining were performed as reported (Guo et al., 2004).

### Histological analysis and confocal microscopy

Embryo or tissue samples were fixed in 10% paraformaldehyde at 4°C overnight, and then dehydrated in a graded series of ethanol, followed by xylene infiltration and paraffin embedding. For cryoembedding, samples were fixed with 2% paraformaldehyde, followed by immersion in a 10-30% graded series of sucrose solution and then embedding in OCT. The paraffin blocks were sectioned at 9 μm using a Leica RM2235 rotary microtome. The cryoembedded specimens were sectioned at 12 μm using a Leica CM 1950.

For phalloidin staining, hearts from E13.5 embryos were cryoembedded, sectioned and stained with phalloidin and antibodies against MF20 (Hybridoma Bank, 1:40). Paraffin sections of the E10.5 embryos were incubated overnight at 4°C with the following antibodies: E-Cadherin (BD Transduction Laboratories, 1:100; San Jose, CA, USA), Laminin (Sigma-Aldrich, 1:200; St. Louis, MO, USA), Beta Catenin (BD Transduction Laboratories, 1:200), Protein Kinase C-zeta (Santa Cruz, 1:200; Dallas, TX, USA), Scrib-11048 (Santa Cruz, 1:100), Vangl2 (R&D System, 1:200; Minneapolis, MN, USA), and Prickle1 (Bassuk et al., 2008). In addition, the biliary ducts were stained with Beta-Catenin and E-Cadherin (BD Transduction Laboratories). Secondary antibodies used included goat anti-mouse Alexa Fluor 488, donkey anti-sheep Alexa Fluor 488, donkey anti-rabbit Alexa Fluor A555, and donkey anti-mouse A555 (all 1:1000). The immunostained sections were imaged using either an Olympus Fluoview Fv1000 or Nikon Eclipse Ti inverted laser scanning confocal microscope.

### Quantitative RT-PCR

Total RNA was isolated from MEFs and the base of the outflow tract of Prickle1 mutant embryos using the RNeasy Plus Mini Kit (Qiagen; Valencia, CA, USA). RNA was reverse transcribed into cDNA using High Capacity RNA-to-cDNA™ (Life Technologies; Grand Island, NY, USA) and qPCR was perform for several canonical and non-canonical WNT genes with β-Actin or GAPDH serving as internal control using the 7900HT Fast Real-Time PCR System (Life Technologies).

### Whole-mount β-gal staining

For *in situ* whole-mount X-gal staining detection of *lacZ* expression via β-gal activity, embryos were fixed with 2% paraformaldehyde, 0.2% glutaraldehyde, and 0.02% Tween-20 in PBS for 1 h at 4°C. After several washes in PBS, whole embryos were incubated in a solution containing 40 mg/ml X-gal (5-bromo-4-chloro-3-indolyl-13-D-galactopyranoside), 20 mM potassium ferricyanide, 20 mM potassium ferrocyanide, 2 mM MgCI_2_, and 10% Tween-20 in PBS. After overnight incubation at 37°C, the embryos were washed with PBT (PBS and 0.1% Tween-20) once for 10 min. Then the stained embryos were examined and imaged using a Leica stereomicroscope.

### Scratch wound assay for polarized cell migration

MEFs were cultured to confluence on 4 well chamber slides and a wound was generated using a 20 μl micropipette tip. Time lapse imaging was carried out using a 20× objective on an inverted microscope (Leica, DMIRE2; Buffalo Grove, IL, USA) with images captures every 10 min for 24 h using a Hamamatsu camera. To observe cell polarity, cells were fixed and immunostained with DAPI and Golgin subfamily A member 2 (GOLGA2; Sigma-Aldrich, 1:1000). GOLGA2 stains the Golgi apparatus in which is situated towards the direction of cell migration having Golgi situated in the front of the nucleus (forward facing) and aligned with migration direction. Polarity was scored as those cells with Golgi oriented within a 0°-180° sector along the direction of wound closure.

### Statistical methods

All continuous data was analyzed for normal distribution skewness and kurtosis normality test. Data that was normally distributed was compared using the Student's *t*-test or one-way analysis of variance for two or more than two groups, respectively. Non-normally distributed data was compared using the Wilcoxon rank-sum test. A two-tailed *P*-value of <0.05 was considered significant. All analyses were performed using Stata 12.0 (StataCorp, College Station, Texas).

### Cilia analysis in the mouse tracheal epithelia

Trachea was freshly harvested from newborn mice and opened longitudinally to expose the ciliated epithelia. The tissue was mounted for video microscopy to assess ciliary beat frequency and motility using previously described methods (Francis and Lo, 2013). For trachea cilia ultra structural analysis, transmission electron microscopy (TEM) and scanning electron microscopy (SEM) (Assemat et al., 2008) were carried out as reported (Vladar et al., 2012). Briefly, adult tracheas from mice of either gender were harvested and fixed in a solution containing 2% glutaraldehyde, 4% paraformaldehyde in 0.1 M Na Cacodylate buffer, pH 7.4 (Electron Microscopy Sciences) at 4°C overnight. Proximal airway direction was tracked throughout the procedure. Samples were osmicated, stained with uranyl acetate, then dehydrated with a graded ethanol series and infiltrated with EMbed-812 (Electron Microscopy Sciences; Hatfield, PA, USA). 80-100 nm sections were mounted onto copper grids and analyzed with a JEOL JEM-1400 microscope (Peabody, MA, USA) using a Gatan Orius Camera (Pleasanton, CA, UAS). For scanning electron microscopy (Assemat et al., 2008), longitudinally opened tracheal segments were fixed as for TEM, osmicated, dehydrated, dried with a Tousimis Autosamdri-815 critical point dryer, then tracheas were mounted luminal side up, sputter coated with 100 Å layer of Au/Pd and analyzed with a Hitachi S-3400N VP-SEM (New York, NY, USA) microscope operated at 10-15 kV, with a working distance of 7-10 mm and using secondary electron detection.

## Supplementary information

Supplementary information available online at http://bio.biologists.org/lookup/suppl/doi:10.1242/bio.015750/-/DC1
